# Exploring the Genomic Patterns in Human and Mouse Cerebellums Via Single-Cell Sequencing and Machine Learning Method

**DOI:** 10.3389/fgene.2022.857851

**Published:** 2022-03-04

**Authors:** ZhanDong Li, Deling Wang, HuiPing Liao, ShiQi Zhang, Wei Guo, Lei Chen, Lin Lu, Tao Huang, Yu-Dong Cai

**Affiliations:** ^1^ College of Food Engineering, Jilin Engineering Normal University, Changchun, China; ^2^ Department of Radiology, State Key Laboratory of Oncology in South China, Collaborative Innovation Center for Cancer Medicine, Sun Yat-sen University Cancer Center, Guangzhou, China; ^3^ Eye Institute of Shandong University of Traditional Chinese Medicine, Jinan, China; ^4^ Department of Biostatistics, University of Copenhagen, Copenhagen, Denmark; ^5^ Key Laboratory of Stem Cell Biology, Shanghai Jiao Tong University School of Medicine (SJTUSM) & Shanghai Institutes for Biological Sciences (SIBS), Chinese Academy of Sciences (CAS), Shanghai, China; ^6^ College of Information Engineering, Shanghai Maritime University, Shanghai, China; ^7^ Department of Radiology, Columbia University Medical Center, New York, NY, United States; ^8^ Bio-Med Big Data Center, CAS Key Laboratory of Computational Biology, Shanghai Institute of Nutrition and Health, University of Chinese Academy of Sciences, Chinese Academy of Sciences, Shanghai, China; ^9^ CAS Key Laboratory of Tissue Microenvironment and Tumor, Shanghai Institute of Nutrition and Health, University of Chinese Academy of Sciences, Chinese Academy of Sciences, Shanghai, China; ^10^ School of Life Sciences, Shanghai University, Shanghai, China

**Keywords:** cerebellum, golgi cells, granule cells, interneuron cells, unipolar brush cells, gene expression pattern, machine learning method

## Abstract

In mammals, the cerebellum plays an important role in movement control. Cellular research reveals that the cerebellum involves a variety of sub-cell types, including Golgi, granule, interneuron, and unipolar brush cells. The functional characteristics of cerebellar cells exhibit considerable differences among diverse mammalian species, reflecting a potential development and evolution of nervous system. In this study, we aimed to recognize the transcriptional differences between human and mouse cerebellum in four cerebellar sub-cell types by using single-cell sequencing data and machine learning methods. A total of 321,387 single-cell sequencing data were used. The 321,387 cells included 4 cell types, i.e., Golgi (5,048, 1.57%), granule (250,307, 77.88%), interneuron (60,526, 18.83%), and unipolar brush (5,506, 1.72%) cells. Our results showed that by using gene expression profiles as features, the optimal classification model could achieve very high even perfect performance for Golgi, granule, interneuron, and unipolar brush cells, respectively, suggesting a remarkable difference between the genomic profiles of human and mouse. Furthermore, a group of related genes and rules contributing to the classification was identified, which might provide helpful information for deepening the understanding of cerebellar cell heterogeneity and evolution.

## Introduction

The cerebellum is like a big regulator and works by affecting the functions of brain, brainstem, and spinal cord at different levels (D'Angelo, 2018). The cerebellum can regulate body balance, muscle tone, and coordination of voluntary movement. An abnormal cerebellum is linked to some neurological diseases, such as autism, schizophrenia, and depression (D'Angelo, 2010; D'Angelo and Casali, 2012). Cellular research reveals the electrophysiological properties of neurons and synapses in the cerebellum and the mechanism of cerebellar synaptic plasticity (Hansel et al., 2001; D'Angelo and De Zeeuw, 2009; D'Angelo, 2011). The heterogeneity of cerebellum cells among different mammalian species presents a species-specific functional pattern of cerebellum which may be linked to evolution. The physiological function of the cerebellum is crucial and it is essential to explore the gene expression of various cells in the cerebellum for understanding its development, evolution, and working mechanism.

The cerebellum is thought to consist of Golgi cells, granule cells (GCs), interneuron cells, and unipolar brush cells (UBC). Cerebellar Golgi cells can receive dual excitatory signals, one of which comes from the mossy fibers of basal dendrites, and the other comes from the parallel fibers of apical dendrites. Golgi cells are inhibitory, and studies showed that the granular layer organization relies on feedforward and feedback inhibition cycles (Eccles Jc Fau - Llinás et al., 1966; Strick, 1985). The anatomical studies of neurons showed that Golgi cells can produce lateral inhibition, which extends beyond the synaptic field. These findings indicate that Golgi cells may regulate the activity of the granular layer. Notably, Golgi cells are regarded as theta-frequency pacemakers activated by localized input bursts, which exploit membrane mechanisms (including specific ionic channels, excitatory, inhibitory chemical synapses, and dendritic gap junctions). Local input pulses activate Golgi cells through membrane mechanisms, such as specific ion channels, synapses, and dendritic gap junctions. GCs are cell types with the highest proportion in the cerebellum and originate from the rhomboid labrum on the dorsal part of the hindbrain alar. GCs constitute the dense and unique structure of the cerebellar cortex (Jaarsma et al., 1996). GCs and Golgi cells are located in the innermost granular cell layer of the cerebellar cortex (Dino et al., 2001; D'Angelo, 2018). Researchers used single-cell transcriptomics methods to reveal the diversity and conservation of granular cells in mice (Jaarsma et al., 1996). Interneuron cells are only a minority in the brain but have the biggest differences in morphology and physiological characteristics (Kepecs and Fishell, 2014). UBC is a glutamatergic neuron located in the cerebellar cortex (Dino and Mugnaini, 2000; Oertel and Young, 2004). Although UBCs may receive the same signal input as GCs, they have unique morphologies, such as dendritic brushes and large ends of axon branches. In accordance with their chemical phenotype and intrinsic characteristics, unipolar brush neurons can be divided into different subgroups (Jaarsma et al., 1996; Dino and Mugnaini, 2000; Dino et al., 2001; Oertel and Young, 2004; Sekerkova et al., 2005). In the process of organismal evolution, the evolution of important genes occupies a core position (Fukushima and Pollock, 2020). The evolution of important organs, such as hominoid brain, is closely related to changes in gene expression (Kaessmann, 2010; Zhang et al., 2011; Chen et al., 2013; Zhang and Long, 2014). Researchers reported that some new genes participated in lineage- or species-specific phenotypic evolution (Chen et al., 2013). In-depth research on human-specific or polymorphic genes may provide important references for exploring the evolution of new genes and their effects on diseases (Cooper and Kehrer-Sawatzki, 2011).

On the basis of existing human and mouse cerebellar cortex single-cell transcriptomic data set (https://www.ncbi.nlm.nih.gov/geo/query/acc.cgi?acc=GSE165371), we use new computational methods to screen for characteristically expressed and important genes between human and mouse cerebellar cells that may affect the development and evolution of the central nervous system. We classified cerebellar cells into four different subtypes including Golgi cell, GC, interneuron cell and UBC. We have built and verified some classifiers that can identify key genes related to species-specific expression pattern and the potential evolutional trend in each cerebellar cell type. We use minimum redundancy and maximum relevance (mRMR) (Peng et al., 2005) combined with incremental feature selection (IFS) (Liu and Setiono, 1998), decision tree (DT) (Safavian and Landgrebe, 1991), random forest (RF) (Breiman, 2001), and Synthetic Minority Oversampling Technique (SMOTE) (Chawla et al., 2002) approaches to recognize the most important gene features and rank these genes based on their relevance in classification (Peng et al., 2005). At the same time, the decision rules for classifying human and mouse cerebellum cells are determined. The candidate feature list contains many meaningful genes, which may play a non-negligible role in the development of the nervous system and the differentiation of nerve cells. Some of the selected features have been confirmed in experiments. On the one hand, this study proves the feasibility and reliability of the analysis methods. On the other hand, selected features provide a direction for further research on the detailed mechanism of nervous system development and pathogenesis and intervention targets of central nervous system diseases.

## Materials and Methods

**FIGURE 1:**
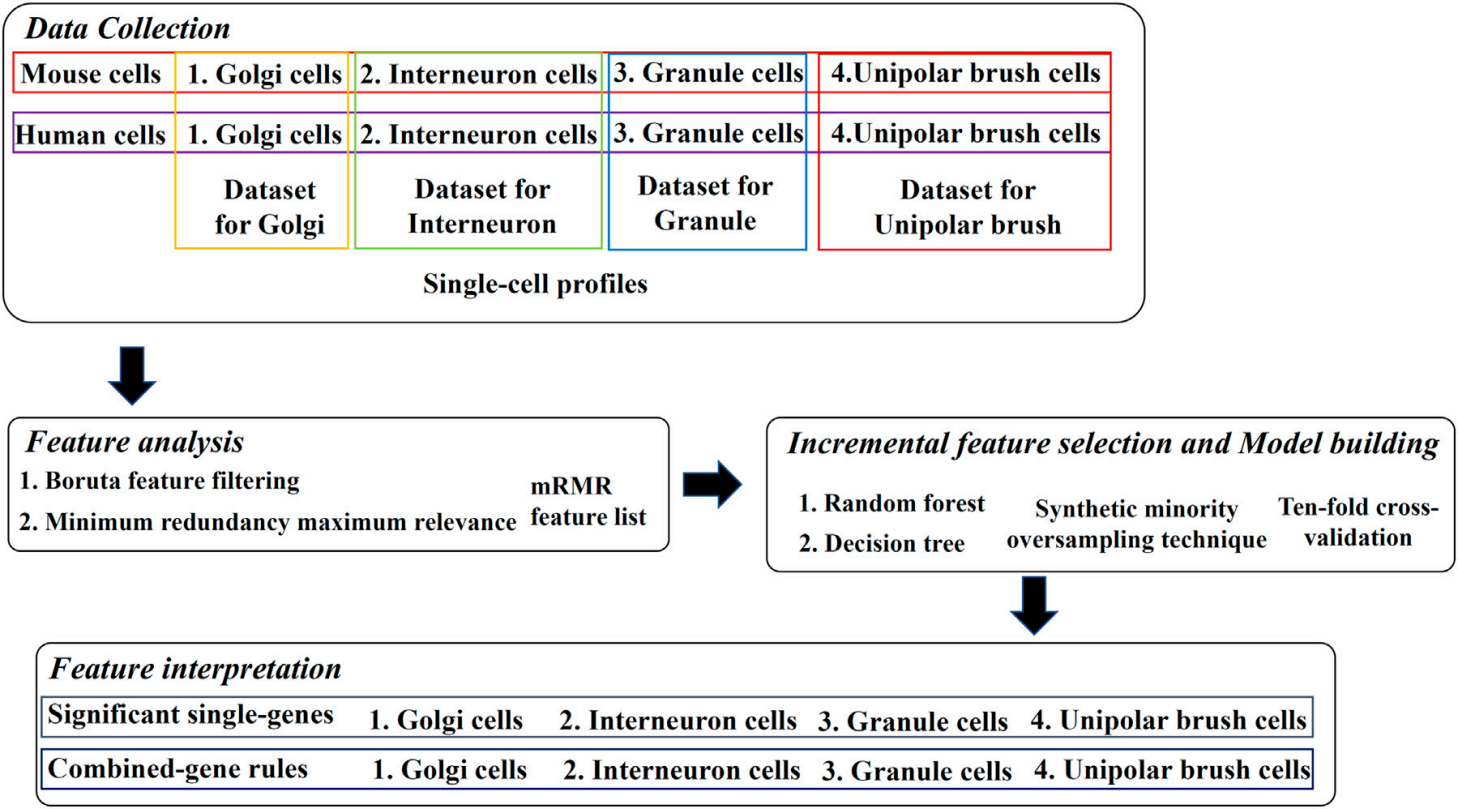
Overview of the design. Four types of mouse and human cerebellum cells constitute four datasets, where cells are represented by single-cell profiles. The profiles are analyzed by Boruta and minimum redundancy maximum relevance feature selection methods one by one, resulting in one mRMR feature list on each dataset. The list is used in the incremental feature selection, incorporating some classification algorithms, synthetic minority oversampling technique and ten-fold cross-validation to extract significant single-genes and combined-gene rules.

Our research is divided into four parts: 1. data collection, 2. feature analysis, 3. incremental feature selection and model building, and 4. feature interpretation. The process is shown in Figure 1, and details are described below.

### Data Collection

Single-cell profiling datasets are downloaded from the transcriptomic atlas of human and mice (https://www.ncbi.nlm.nih.gov/geo/query/acc.cgi?acc=GSE165371). The total numbers of samples and features in the dataset are 321,387 and 74,593, respectively, which are composed of four different cell sample datasets, corresponding to four sell types: Golgi cell, GC, interneuron cell, and UBC. The breakdown of each dataset, including number of mouse cells, number of human cells, total number of cells and number of gene features, is provided in Table 1. A binary classification problem was employed to investigate each dataset, where mouse cells were termed as positive samples and human cells were considered as negative samples.

**TABLE 1:** Breakdown of 4 cell sample datasets.

Cell type	Number of mouse cells	Number of human cells	Total number of cells	Number of gene features
Golgi cell	3,989	1,059	5,048	14,512
Granule cell	119,972	130,335	250,307	23,422
Interneuron cell	45,555	14,971	60,526	23,203
Unipolar brush cell	1,613	3,893	5,506	13,456

### Feature Analysis

#### Boruta Feature Filtering

The Boruta feature selection is a RF-based wrapper method used to detect all relevant features related to the target output (Kursa and Rudnicki, 2010; Zhang et al., 2021a) and identifies related features by iteratively identifying the important scores of real and shuffled features. Specifically, the Boruta feature selection copies the training dataset and scrambles the value of the feature to obtain a new dataset called the shuffled dataset. The RF classifier is trained on this shuffled dataset to obtain the importance score of each feature. The real feature with a remarkably higher importance score than the shuffled feature is marked as important. These important features are selected by Boruta after multiple iterations.

The Boruta program used in this study is retrieved from https://github.com/scikitlearn-contrib/boruta_py. It is performed with its default parameters. Features selected by Boruta are further investigated by another feature selection method.

#### Minimum Redundancy and Maximum Relevance

The mRMR (Peng et al., 2005; Zhang et al., 2021a; Pan et al., 2021; Chen et al., 2022) is a feature selection method used to determine the relationship between features and classification predictions. The mRMR can calculate the feature relevance between features and labels as well as the redundancy of features through filters and obtain the optimal subset by ranking the features with high feature relevance and low feature redundancy. A feature list, named mRMR feature list, is generated by mRMR. Initially, this list is empty. mRMR repeatedly selects one feature from the remaining features such that it has highest relevance to labels and lowest redundancy to already-selected features. This selected feature is appended to the list. The procedure stops until all features are in the list.

This study adopts the mRMR program obtained from http://penglab.janelia.org/proj/mRMR/. Default parameters are used to perform such program.

### Incremental Feature Selection and Model Building

Although mRMR produces a feature list, users still cannot know which features should be selected for model building. In our study, such procedure is fulfilled by the integration of the IFS (Liu and Setiono, 1998) approach with RF (Breiman, 2001) and DT (Safavian and Landgrebe, 1991) algorithms.

The IFS (Liu and Setiono, 1998) is a feature selection approach that aims to select optimal features for the creation of a supervised classifier. To perform IFS on a descending list of features, we first construct a series of feature subsets, each of which contains some top features in the list. On each feature subset, a classifier is built and its performance is evaluated by ten-fold cross-validation (Kohavi, 1995; Ding et al., 2022; Tang and Chen, 2022). After testing all possible feature subsets, the classifier with highest performance is discovered. The features for such classifier is the optimal features, and the classifier is the optimal classifier.

As IFS method needs one classification algorithm, we employ two classic algorithms in this study. They are RF and DT. The RF is a meta-classifier containing a large number of DTs, where each DT is built based on randomly selected samples and each node in such DT computes the output through a random subset of features. The outputs of the DTs are aggregated to generate the final output class. RF is quite powerful. Thus, it has wide applications in tackling many biological and medical problems (Bifsha et al., 2014; Zhao et al., 2018; Jia et al., 2020; Liang et al., 2020; Liu et al., 2021; Pan et al., 2021; Li et al., 2022; Yang and Chen, 2022). RF reduces errors by averaging the predicted outputs of all DTs because of some variations between DTs. This phenomenon loses some interpretability, slightly increases bias, improves performance, and avoids overfitting.

Different from the RF algorithms, which acts as a kind of “black-box” classifier, DT can construct classification and regression models that are understandable by humans. Although it is not very powerful, it can provide novel insights to uncover underlying mechanism. This algorithm has also been used to deal with some important problems (Liang et al., 2020; Zhang et al., 2021a; Chen et al., 2021; Pan et al., 2021). The tree generated by DT generally consists of a set of interpretative rules, indicating the contributive roles of features to the final model in the format of “IF–THEN” conditions.

In our study, RF and DT are implemented using the Scikit-learn package in *Python*.

### Synthetic Minority Oversampling Technique

As listed in Table 1, four datasets are imbalanced. Models directly built on such datasets are always not efficient. Here, we adopt SMOTE (Chawla et al., 2002) to process this problem, which is a technique for oversampling based on the principle of creating synthetic data by using the k-nearest neighbor algorithm. First, a sample, denoted by *x*, is randomly selected from the minor class. Second, the k-nearest neighbors of *x*, which are also in the minor class, are found, and one neighbor is randomly selected, denoted as *y*. Third, a synthetic sample is created by *x* and *y*, which is defined as the linear combination of *x* and *y* with randomly generated combination coefficients. This sample is poured into the minor class. Such procedures are executed several times until minor class has equal number of samples to the major class. In this study, SMOTE is only used in the evaluation of classifiers in IFS method.

### Feature Interpretation

In our study, several machine algorithms are applied on four datasets of cerebellum cells. We can obtain some essential gene features. The interpretation of gene features includes two parts, i.e., interpretations of single- and combined-gene rules. The single-gene interpretation focuses on the optimal gene selected by the mRMR and IFS, whereas the interpretation of the combined-gene rule focuses on the predictive rules given by DT. Our interpretation is based on a comprehensive literature review of a previous work.

### Performance Evaluation

The Matthew’s correlation coefficient (MCC) (Matthews, 1975; Chen et al., 2017; Zhao et al., 2018; Jia et al., 2020; Liang et al., 2020; Zhang et al., 2021b) is used to evaluate the performance of training models. MCC is the correlation coefficient between the observed categories and predictions. MCC serves as an indicator that can be applied to samples with large imbalance. MCC is defined as:

$$MCC=\frac{TP\times TN-FP\times FN}{\sqrt{\left(TP+FP\right)\left(TP+FN\right)\left(NP+FP\right)\left(NP+FN\right)}}$$ (1)

where TP, FP, TN, and FN represent the numbers of true-positive, false-positive, true-negative, and false-negative samples, respectively. The value range of MCC is distributed between −1 and 1. A high MCC indicates good performance of the classifier.

In additional, we also employ other measurements, including sensitivity (SN) (same as recall), specificity (SP), prediction accuracy (ACC), precision and F1-measure. They can be computed by

$$SN=\frac{TP}{TP+FN}$$ (2)

$$SP=\frac{TN}{TN+FP}$$ (3)

$$precision=\frac{TP}{TP+FP}$$ (4)

$$F1-measure=\frac{2\times recall\times precision}{recall+precision}$$ (5)

These measurements are provided for reference.

## Results

### Data Collection and Feature Analysis

In our study, 321,387 cerebellum cell samples were collected. These single-cell samples could be divided into four categories, i.e., Golgi cell (5,048, 1.57%), GC (250,307, 77.88%), interneuron cell (60,526, 18.83%), and UBC (5,506, 1.72%). In the original cell sample data, each cell sample was represented by expressions on lots of genes, which were quite difficult for machine learning analysis due to the dimensionality curse. Therefore, the Boruta filtering method was first used to do the optimal compression of features (i.e., gene expressions) for each of 4 cell datasets. After compression, each Golgi cell, GC, interneuron cell, and UBC was represented as a compressed vector of 1,276, 1,271, 1924, and 1,252 features, respectively. The gene ID corresponding to the feature in the sample vector could be found in Supplementary Table S1.

Remaining features were analyzed by the mRMR method. An mRMR feature list was obtained for each dataset. Four feature lists are also provided in Supplementary Table S1. These lists were further investigated in the following procedures.

### Prediction Performance

**FIGURE 2:**
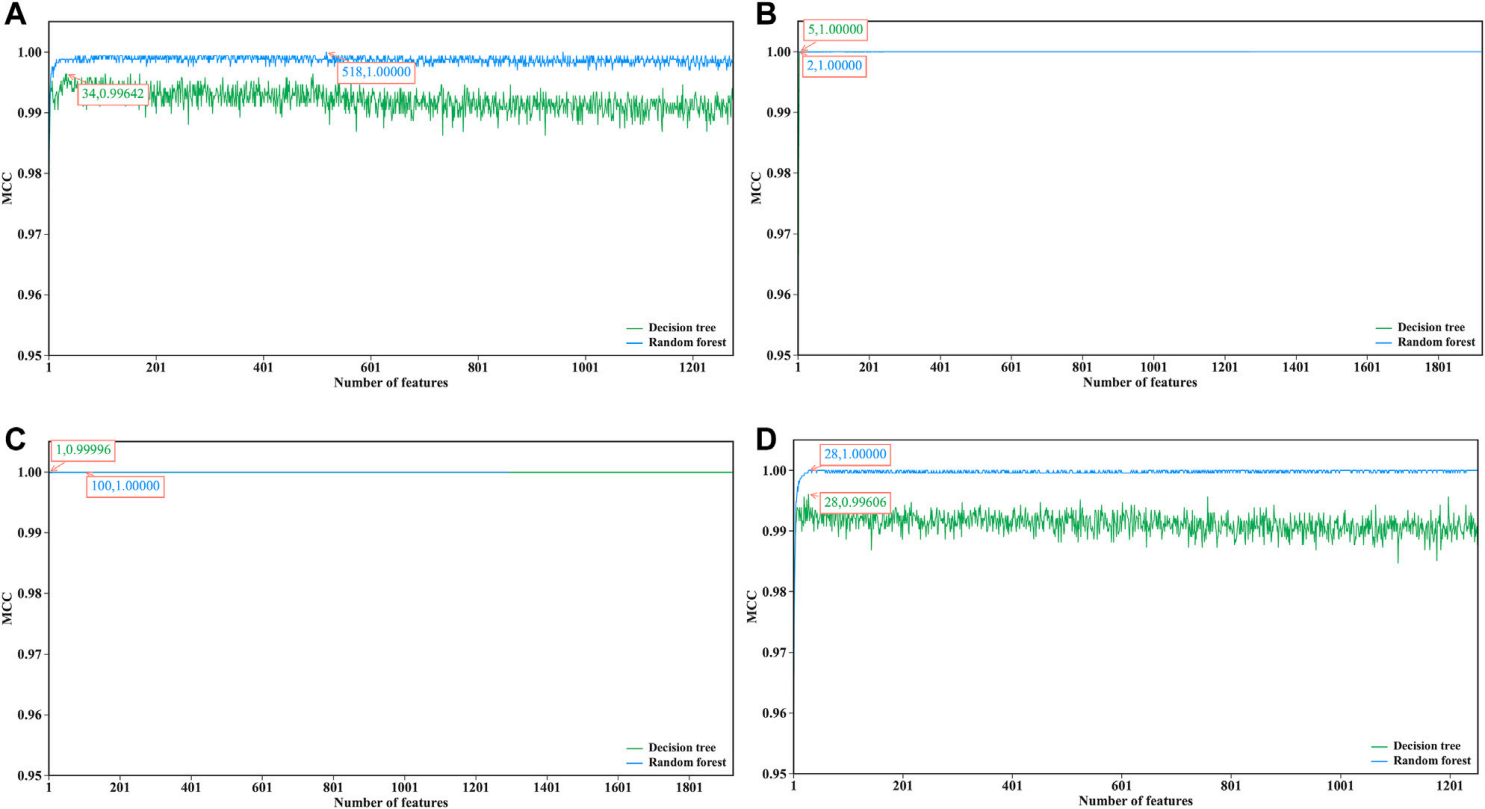
IFS curves of decision tree and random forest on datasets of four cerebellum cell types. **(A)** Curves on dataset for Golgi cells, **(B)** Curves on dataset for Granule cells, **(C)** Curves on datasets for Interneuron cells, **(D)** Curves on dataset on Unipolar brush cells.

For each dataset, an mRMR feature list was obtained. Afterward, RF and DT classification algorithms were used to construct the classification models in the IFS method with a step size of one on the basis of such list. Each classification model was evaluated by ten-fold cross-validation. The predicted results were counted as measurements listed in *Performance Evaluation* section. To clearly show the performance of DT and RF on different feature subsets, an IFS curve was plotted for each classification algorithm, as shown in Figure 2, where *x*-axis represents the number of features in the subset and *y*-axis stands for the main measurement, MCC. The key information extracted from these IFS curves is listed in Table 2.

**TABLE 2:** Performance of optimal classifiers on four datasets using different classification algorithms.

Cell type	Classification algorithm	Number of features	MCC
Golgi cell	Decision tree	34	0.99642
	Random forest	518	1.00000
Granule cell	Decision tree	5	1.00000
	Random forest	2	1.00000
Interneuron cell	Decision tree	1	0.99996
	Random forest	100	1.00000
Unipolar brush cell	Decision tree	28	0.99606
	Random forest	28	1.00000

**FIGURE 3:**
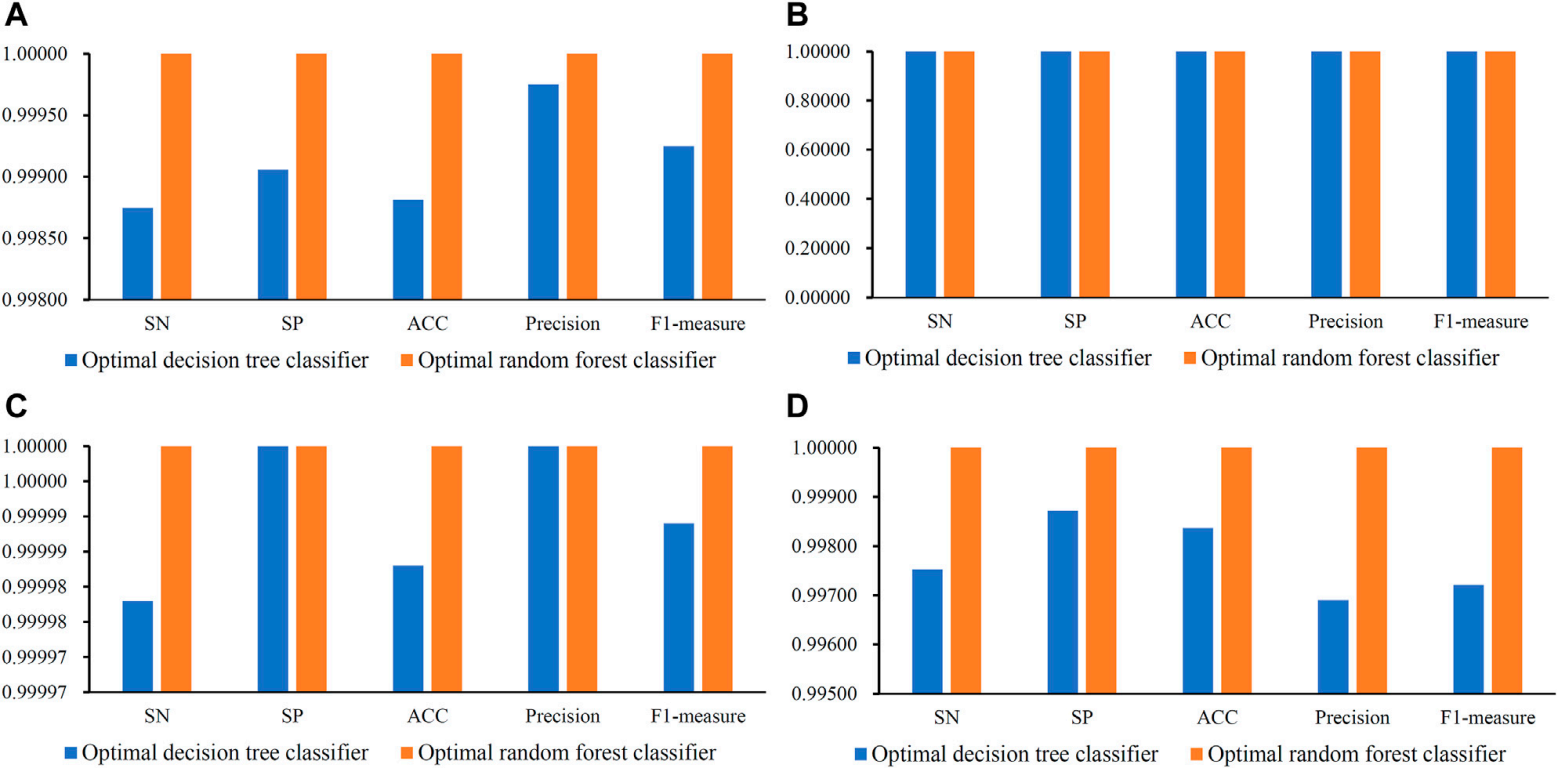
Some measurements of the optimal decision tree and random forest classifiers on datasets of four cerebellum cell types. **(A)** Measurements on dataset for Granule cells, **(B)** Measurements on datasets for Granule cells, **(C)** Measurements on datasets for Interneuron cells, **(D)** Measurements on datasets for Unipolar brush cells.

When RF was used in the IFS method, it achieved perfect performance with MCC = 1.00000 when proper feature subsets were adopted. In detail, for Golgi cell, 518 top features in the mRMR feature list were used, whereas this number was 2, 100 and 28 for other three types of cells, respectively. These features constituted the optimal features for each dataset. Accordingly, an optimal RF classifier was built on each dataset using corresponding optimal features. Their detailed performance, including SN, SP, ACC, precision and F1-measure, is illustrated in Figure 3. Evidently, each measurement reached its perfect value, suggesting extreme good performance of these classifiers. They can be efficient tools to classify mouse and human cerebellum cells.

Although the optimal RF classifiers yielded perfect performance, they cannot provide useful clues to uncover essential differences between mouse and human cerebellum cells because RF is a black-box algorithm. In view of this, DT was employed in this study, which can provide more clear insights to study mouse and human cerebellum cells. It can be observed from Figure 2 that the highest MCC on each dataset yielded by DT was also very high (>0.99000). These MCC values were obtained by using top 34, 5, 1 and 28 features in the list on four datasets, respectively. Accordingly, these top features comprised the optimal features for four datasets, respectively, and an optimal DT classifier with corresponding optimal features was built on each dataset. Other measurements of these optimal DT classifiers are shown in Figure 3. Clearly, on each dataset, the optimal RF classifier was always superior to or equal to the optimal DT classifier. This result conformed to the general fact that RF is more efficient than DT.

### Effectiveness of the Optimal Classifiers

**FIGURE 4:**
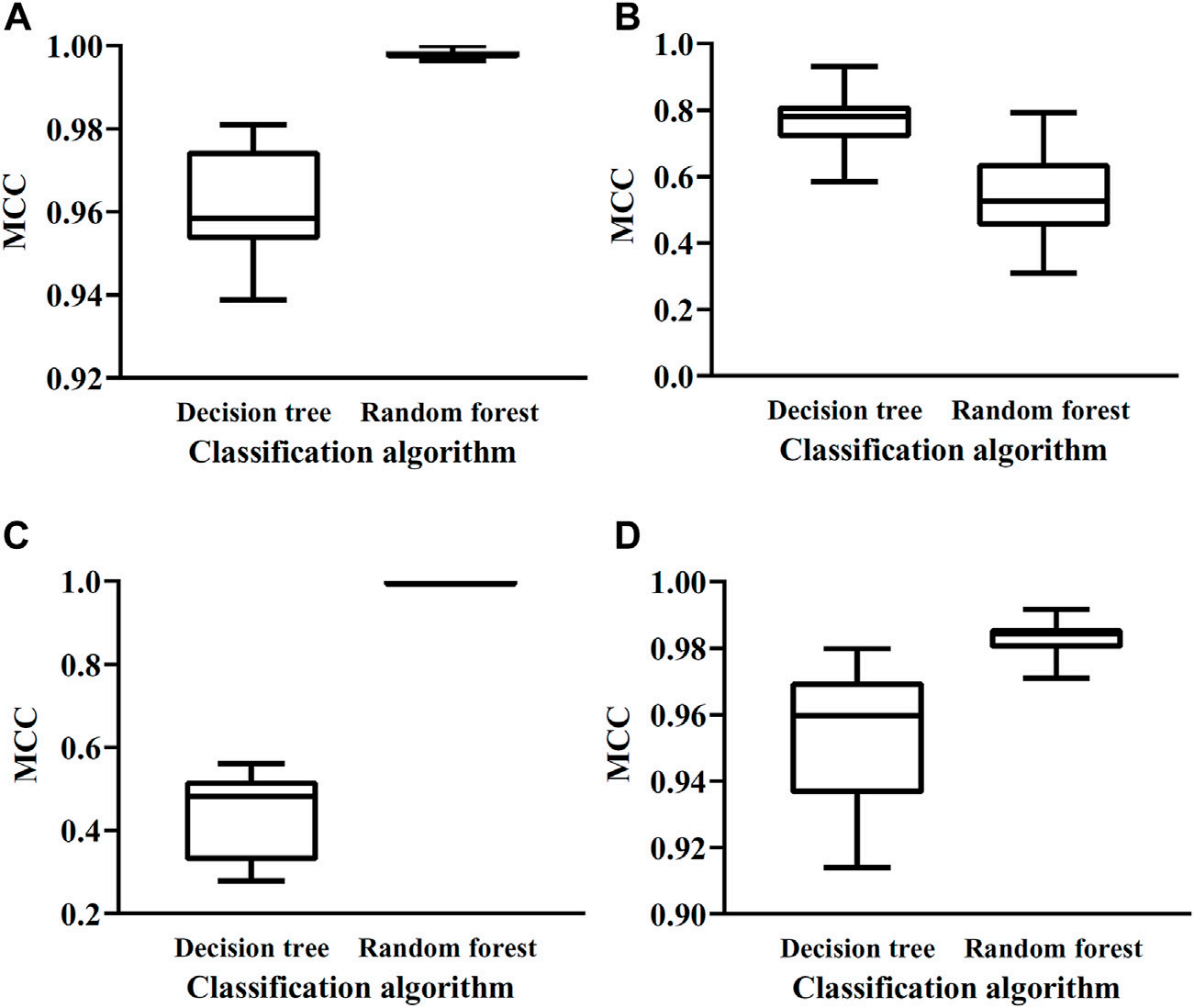
Box plots of MCC values yielded by classifiers with randomly selected gene features on datasets of four cerebellum cell types. **(A)** Box plots on dataset for Granule cells, **(B)** Box plots on datasets for Granule cells, **(C)** Box plots on datasets for Interneuron cells, **(D)** Box plots on datasets for Unipolar brush cells.

The optimal DT/RF classifiers constructed above shown good even perfect performance. This section elaborated that these results were not incidental. To this end, for each optimal classifier, we did the following test. According to the number of features in the optimal classifier, i.e., the number of optimal features, we randomly selected same number of features from all features. These selected features were used to represent mouse and human cerebellum cells. A classifier with DT or RF was built on such representation. Ten-fold cross-validation was employed to evaluate its performance. To give a full test, above procedures were conducted twenty times. Obtained MCC values were shown in a box plot, as illustrated Figure 4. It can be observed that these classifiers with randomly selected features all provide lower performance than the corresponding optimal classifier. This indicated that the optimal features were really important for classifying mouse and human cerebellum cells and can be significant single-genes to distinguish mouse and human cerebellum cells.

### Significant Feature Interaction

Important genes for distinguishing human and mouse cerebellar cells based on the mRMR ranking are presented in Table 3. These genes can be significant single-genes to distinguish mouse and human cerebellum cells, which would be discussed in *Analysis of Significant Single-Genes* section.

**TABLE 3:** Feature list of important genes based on mRMR ranking.

Cell type	The rankings of feature	Genes
Golgi Cell	1	Lingo2
	3	ube3a
	5	Nlgn1
Granule Cell	1	Ralyl
	3	Fgf14
Interneuron Cell	1	Malat1
	2	Ank2
	3	Nrxn3
Unipolar Brush Cell	1	Pde1a
	7	Rgs6

Furthermore, we adopted the rule learning algorithm DT to generate combined-gene rules, interpret the classification rules of features. According to the optimal DT classifier on each dataset, a tree was learnt on all cell samples, which were represented by the optimal features of this DT classifier. Such tree was represented by some rules, which are listed in Table 4. A total of 8, 2, 2, and 11 rules were obtained for the Golgi cell, GC, interneuron cell, and UBC, respectively. In *Analysis of Combined-Gene Rules* section, we would discuss these rules.

**TABLE 4:** Classification rules generated by DT.

Index	Rule		Label
Golgi Cell gene			
1	(Lingo2 > 2,924.455) and (Lrp1b ≤ 1,185.155) and (Upk3b ≤ 180.656)		Negative
2	(Lingo2 ≤ 2,924.455) and (Pla2g3 ≤ 108.329) and (Nrxn1 ≤ 5,364.268) and (Ube3a ≤2,781.385)		Positive
3	(Lingo2 ≤ 2,924.455) and (Pla2g3 > 108.329)		Negative
4	(Lingo2 > 2,924.455) and (Thsd7b > 1,185.155)		Positive
5	(Lingo2 ≤ 2,924.455) and (Pla2g3 ≤ 108.329) and (Nrxn1 ≤ 5,364.268) and (Ube3a >2,781.385) and (Fstl5 ≤ 539.2883)		Negative
6	(Lingo2 ≤ 2,924.455) and (Pla2g3 ≤ 108.329) and (Nrxn1 > 5,364.268)		Negative
7	(Lingo2 ≤ 2,924.455) and (Pla2g3 ≤ 108.329) and (Nrxn1 ≤ 5,364.268) and (Ube3a >2,781.385) and (Fstl5 > 539.288)		Positive
8	(Lingo2 > 2,924.455) and (Thsd7b ≤ 1,185.155) and (Upk3b > 180.656)		Positive
Granule Cell gene			
1	Malat1 ≤ 3,654.637		Positive
2	Malat1 > 3,654.637		Negative
Interneuron Cell gene			
1	Malat1 > 945.180		Negative
2	Malat1 ≤ 945.1805		Positive
Unipolar Brush Cell gene			
1		(Ccdc85a ≤152.189) and (Rgs6 ≤ 4,444.267) and (Kcnd2 ≤ 5,474.120) and (Cdh12 ≤ 1975.607) and (Fgf14 ≤ 7,328.168)	Positive
2		(Ccdc85a >152.189) and (Hsp90aa1≤1,532.738)	Negative
3		(Ccdc85a≤152.189) and (Rgs6 > 4,444.267) and (Aff3≤1,376.276)	Negative
4		(Ccdc85a≤152.189) and (Rgs6≤4,444.267) and (Kcnd2 > 5,474.120) and (Cblb≤284.311)	Negative
5		(Ccdc85a≤152.189) and (Rgs6≤4,444.267) and (Kcnd2≤5,474.120) and (Cdh12≤1975.607) and (Fgf14 > 7,328.168) and (Kcnd2≤2,874.838)	Positive
6		(Ccdc85a >152.190) and (Hsp90aa1 > 1,532.738)	Positive
7		(Ccdc85a≤152.189) and (Rgs6 > 4,444.267) and (Aff3 > 1,376.276)	Positive
8		(Ccdc85a≤152.189) and (Rgs6≤4,444.267) and (Kcnd2≤5,474.120) and (Cdh12≤1975.607) and (Fgf14 > 7,328.168) and (Kcnd2 > 2,874.838)	Negative
9		(Ccdc85a≤152.189) and (Rgs6≤4,444.267) and (Kcnd2 > 5,474.120) and (Cblb >284.311)	Positive
10		(Ccdc85a≤152.189) and (Rgs6≤4,444.267) and (Kcnd2≤5,474.120) and (Cdh12 > 1975.607) and (Pde1a >2,364.066)	Positive
11		(Ccdc85a≤152.189) and (Rgs6≤4,444.267) and (Kcnd2≤5,474.120) and (Cdh12 > 1975.607) and (Pde1a≤2,364.066)	Negative

## Discussion

In this project, we used machine learning methods to explore the single-cell expression profile data of human and mouse cerebellar cells. Important single-genes and combined-gene rules of each cerebellar cell type for distinguishing these two mammalian species are created and shown in Tables 3, 4. The classification achieved a quite high accuracy that indicates a considerable difference in expression pattern between human and mouse cerebellar cells. To further validate the usefulness of our models and understand the functional evolution of cerebellar cells, we summarized existing experimental evidence for the important genes and rules through a wide literature review.

### Analysis of Significant Single-Genes

According to features used in the optimal RF classifiers, we identified 648 (518+2+100+28) considerable features with the mRMR method to distinguish human and mouse cerebellum cells. Next, we further introduced research evidence related to the most important features in each cerebellar cell type to confirm the reliability of previous forecasts.

### Golgi Cell Gene

The protein encoded by leucine-rich repeat and immunoglobulin (Ig) domain containing 2 (*LINGO2*) is identified as a member of the leucine-rich repeats (LRR) gene family. The expression of the *LINGO2* gene in the hypothalamus and cerebral cortex hypothalamus is higher than that in other regions. The Lingo2 is in the top relevant feature with the mRMR method and has been linked with essential tremor (ET) and Parkinson’s syndrome (Wu et al., 2011). ET is the most common movement disorder and adult patient accounts for the vast majoritys. Studies showed that LRR and LINGO2 protein structural variations containing Ig domains may be related to ET. Compared with those of the control group, the protein levels of LINGO1 in the cerebellar cortex and cerebellar white matter of patients with ET are significantly increased. Changes in the LINGO2 expression in the diseased brain appear to occur as the disease progresses, starting in the cerebellar cortex before reaching the white matter. Compared with those of normal individuals, the LINGO1 protein levels in the cerebellar cortex and white matter of patients are significantly increased. In addition, the expression of LINGO2 of patients shows consistent changes with the progression of ET, which starts from cerebellar cortex and then reaches the white matter. The upregulation of *LINGO* expression is likely to be a potential pathological indicator of neurodegenerative diseases (Jasinska-Myga and Wider, 2012; Delay et al., 2014). Studies confirmed that a tSNP variant of *LINGO2* is associated with Parkinson’s syndrome (*p* < 0.05) (Chen et al., 2015). Our results show that LINGO2 is significantly different in the cerebellum of humans and mice, indicating that the LINGO2 expression may be associated with the development of the nervous system. The above research results remind us that the expression level of LINGO2 may be related to the nervous system especially the evolutionary level of cerebellum. In addition, the *LINGO2* mutant may be an important indicator of neurodegenerative diseases.

The protein-coding gene *UBE3A* encodes the E3 ubiquitin protein ligase, which can bind to the ubiquitin of the E2-binding enzyme in the form of a thioester and then transfer ubiquitin to E2-binding enzyme substrate (Kumar et al., 1999; Dhananjayan et al., 2006; Mishra et al., 2009; Shimoji et al., 2009; Gossan et al., 2014; Ronchi et al., 2014). In addition, *UBE3A* can accelerate the degradation of misfolded proteins, thereby achieving cell quality control. The *UBE3A* gene presents a biallelic expression pattern in some tissues, but its transmission mode in the brain is maternal inheritance. The *Ube3A* mutation can cause the Angelman syndrome (Buiting et al., 2016), a neurological disease accompanied by severe developmental delay, hypotonia, epilepsy, aphasia, and other complications. In addition, reports showed that the UBE3A protein binds to the E6 protein of papillomavirus, causing p53 ubiquitination and hydrolysis. Other studies showed that UBE3A can mediate the activity-regulated cytoskeleton-associated protein (ARC) ubiquitination and degradation to regulate synaptic growth (Greer et al., 2010). In addition, mutations in *Ube3A* are related to autism. Researchers found that Ube3A dysfunction can increase ARC expression and reduce the quantity of α-amino-3-hydroxy-5-methyl-4-isoxazolepropionic acid (AMPA) receptors in synapses. Therefore, researchers inferred that AMPA dysregulation may be related to Angelman syndrome or other cognitive disorders (Greer et al., 2010). In our analysis results, *Ube3A*, as one of the top features, has significant differences in expression in human and mouse cerebellum cells. Combined with existing studies, our study suggests that the expression level of Ube3A may serve as a powerful indicator for predicting the function and evolution of the nervous system.

Neuroligin 1 (*NLGN1*) is a protein-coding gene, and its translation product is a member of the neuron cell surface protein family. The NLGN1 family can be used as specific ligands for β-neuroproteins, which may be related to the formation and remodeling of synapses. The NLGN1 protein interacts with neuroproteins to promote synaptic transmission signals and recruits and accumulates other synaptic proteins. Studies showed that *NLGN1* can promote the *de novo* formation of synaptic structures *in vitro* and may participate in the regulation of excitatory synapses. The protein encoded by *NLGN1* has hydrolase and protein dimerization activities and plays an important role in protein–protein interaction at the synapse and signal transmission process across the synapse. *NLGN1* variants may result in autism and Asperger’s syndrome. We speculated that the differential expression pattern of NLGN1 in Golgi cells between human and mouse cerebellum may imply a potential functional evolution.

### Granule Cell Gene

The Raly-like recognition motif (*Ralyl*) is identified as an important characteristic gene by the mRMR, and its encoded RNA binding protein affects embryonic development (Ji et al., 2003a). A previous study pointed out that Ralyl may be related to Alzheimer’s disease (Zhang et al., 2020). Researchers revealed that *Ralyl* is a hub gene in the brain transcriptome module of patients with Alzheimer’s disease and is highly associated with Alzheimer’s reserve-related phenotypes. Notably, the *Ralyl* expression decreases with Alzheimer’s progression. Subjects with Alzheimer’s disease reserves show significantly higher *Ralyl* expression compared with those without Alzheimer’s disease reserves (Zhang et al., 2020). *Ralyl* is related to cancer cell metastasis and poor prognosis in patients with liver cancer. *Ralyl* is specific for liver progenitor cells and regulates hepatocellular carcinoma stem cells by upregulating the stability of TGF-β2 mRNA through the reduced N6-methyladenosine modification (Wang et al., 2021). In addition, the overexpression of *Ralyl* can inhibit the MAPK and CDH1 signaling pathways, thereby inhibiting the development of ovarian cancer (Xia et al., 2021). Compared with those in nontumor tissues and epithelial ovarian cancer cells, the expression level of *Ralyl* in ovarian clear cell cancer cells is lower. The pathological stage and prognosis of patients with ovarian clear cell carcinoma and high Ralyl expression are improved. Other diseases associated with Ralyl include Bardet–Biedl syndrome 1. Ralyl can be regarded as a prognostic marker for certain tumors and a monitoring target for central nervous system disorders.

The protein-coding gene *Fgf14* belongs to the fibroblast growth factor (FGF) family. Members of this family can promote cell mitosis and are closely related to other biological processes. The mutation of this gene is related to autosomal dominant-inherited brain ataxia (Miura et al., 2019). *Fgf14* related pathways include ERK signaling and apoptosis pathways in synovial fibroblasts. FGF14 is an intracellular protein that controls neuronal excitability and synaptic transmission and is suggested for use in the nervous system and mental diseases. Studies showed that male *Fgf14* knockout mice have significantly reduced aggressiveness, sexual behavior, and other behaviors driven by spontaneous initiatives. The fine-tuning of neuronal function by Fgf14 is an important mechanism for controlling such behaviors. FGF14 can control the excitability and synaptic transmission of neurons and has certain diagnostic indicators in neurological and mental diseases. Further molecular studies revealed that Fgf14 can affect individual behaviors by regulating the function of neurons (Hoxha et al., 2019). Recent data indicate that *Fgf14* can modulate multiple ion channels and the localization of the potassium voltage-gated channel subfamily Q member 2 (KCNQ2) protein in hippocampal neurons (Pablo and Pitt, 2017). All these results proved the important role of Fgf14 in regulating nervous functions. Our analysis proposed a new sight that Fgf14 shows differential expression in GCs between human and mouse cerebellum, and implying a linkage between gene Fgf14 and nervous system evolution in mammals.

### Interneuron Cell Gene

NRXN3 belongs to the neuroprotein (NRXN) family, which can act as a cell adhesion molecule in the process of synaptogenesis and intercellular signaling. *NRXN3* has a wide range of alternative splicing and alternative promoters. Thus, the gene has multiple transcription variants and protein isoforms. Previous research suggested that *NRXN3* variants are associated with abnormal behavioral phenotypes, such as alcohol dependence, nicotine addiction, and autism spectrum disorders. However, new research showed that *NRXN3* also plays a potential role in disorders of synaptic transmission. *NRXN3*-related pathways include muscular dystrophy and protein interactions at synapses. Studies showed that mice with missense variants of *NRXN3* show increased fear. The possible change in *NRXN3* from arginine to tryptophan is a pathogenic variant of empathy and fear (Keum et al., 2018). Researchers collected one data set related to healthy aging and 3 data sets related to Alzheimer’s disease in the hippocampus from the Gene Expression Omnibus database. The results of functional analysis showed that NRXN3-led synaptic dysfunction plays a prominent role in the process of aging and Alzheimer’s disease-related cognitive decline. In addition, when the expression of *NRXN3* in an individual decreases, the risk of Alzheimer’s disease increases, but its underlying mechanism needs to be further elucidated (Zheng et al., 2018). *NRXN3* encodes an important part of synaptic function related to autism and other neurodevelopmental/neuropsychiatric diseases (Südhof et al., 2008). The chromosome microarray analysis is used to identify rare exon deletions affecting the *NRXN3* alpha isoform in three-generation Chinese families. The results of family cosegregation studies indicate that NRXN3 affects autism and neurodevelopment/neuropsychiatric disorders. Moreover, schizophrenia and facial deformities are potential new features of NRXN3 haploid deficiency (Yuan et al., 2018). A study showed that the *NRXN3* gene is a potential factor affecting the risk of nicotine addiction and that the NRXN3 marker rs1004212 is significantly related to the amount of smoking (Novak et al., 2009). Combining our analysis results and existing research, we further speculate that the abnormal expression of *NRXN3* is the cause of neurological diseases that cannot be ignored, and its expression level may also be an important marker for representing the evolution of the nervous system of different species.

The protein encoded by Ankyrin 2 (*ANK2*) belongs to the ankyrin family and connects integral membrane proteins with the cytoskeleton. Ankyrin is involved in cell proliferation and movement and the maintenance of special domains. ANK2 can promote the localization of ion transporters and channels and maintain the stability of cell membranes. For example, in cardiomyocytes, ANK2 can coordinate the assembly of ion exchangers to maintain and promote the targeting and stability of ion exchangers. In addition, in neonatal cardiomyocytes, ANK2 is indispensable for regulating the contraction rate. In the skeletal muscle, ANK2 is involved in the correct positioning of DMD and DCTN4 and in the formation and/or stabilization of microtubule subsets related to the ribs and neuromuscular junctions. In the rod-shaped photoreceptor, ANK2 participates in the coordinated expression of Na/K atpase, Na/Ca exchanger, and β-2-spectrin. In addition, ANK2 is involved in important life processes, such as endocytosis and intracellular protein transport. ANK2 variants can cause long QT syndrome four and arrhythmia syndrome (Watanabe and Minamino, 2016; Gessner et al., 2019). The association between gene ANK2 and interneuron cells of cerebellum have not been reported so far. Our study demonstrated the significant difference in ANK2 expression between human and mouse cerebellum, and it suggested a potential role of ANK2 in nervous system development and evolution.

### Unipolar Brush Cells Gene

The phosphodiesterase (PDE) 1A gene encodes a Ca^2+^/calmodulin-dependent PDE, which includes 23 exons and 9 subtypes. The PDE1A gene belongs to the cyclic nucleotide PDE family. PDE, a phosphohydrolase, catalyzes the hydrolysis of adenosine (cAMP) and/or guanine (cGMP) 3′,5′-cyclic phosphate in the 3′-cyclic phosphate bond. PDE1A can regulate the concentration of cyclic nucleotides in the cell and influence signal transduction. The cyclic nucleotide PDE has dual specificity for cAMP and cGMP and is involved in the regulation of some important physiological processes. PDE1A can bind calmodulin and cGMP, has higher affinity for cGMP than for cAMP, and occupies an important position in the GPCR and calcium signaling pathway. Current studies found that PDE1A9 is highly expressed in the brain tissue, but its expression may lead to functional changes depending on age. Studies showed that compared with young controls, the phosphorylation level of the transmembrane regulatory protein in the hippocampus of aged rats is significantly reduced (Kelly et al., 2014), which may be related to the expression of PDE1A in individuals of different ages. Other diseases associated with PDE1A include Fraser’s syndrome 1.

The encoded product of the G protein signal regulator 6 (RGS6) belongs to the G protein signal transduction regulator protein family. The RGS6 protein is characterized by DEP and GGL domains. The latter is the G beta 5 interaction domain, and these proteins can activate the gtpase activity. Many alternatively spliced transcripts of this gene have long or short N-terminal domains, complete or incomplete GGL domains, and isotypes of different C-terminal domains. The RGS protein may regulate G protein-mediated signal transduction through negative feedback, thereby affecting the activity of neurons, cardiovascular system, and lymphocytes and may even increase the risk of cancer. Mutations in *RGS6* may cause Hirschsprung’s disease 1, night blindness and congenital quiescence. RGS6-related pathways include GPCR signal transmission and protein metabolism. When the G protein surface receptor is activated, the G protein initiates a signal cascade in the host cell. The RGS protein inactivates the G protein and turns off this signaling cascade. RGS6 belongs to the R7 subfamily and regulates the G protein function, which is essential for the transmission of a variety of neurotransmitters and neuronal responses. Genetic variations in RGS6 may disrupt normal GPCR signals, leading to disease or subtle features. For example, studies reported that RGS6 abnormalities may be related to diseases, such as alcohol dependence, Parkinson’s syndrome, and neurological or affective disorders (Ahlers et al., 2016). In human dopamine neurons, the expression of RGS6 is restricted but can regulate the D2R-Gi/o pathway and can prevent Parkinson’s neurodegeneration, resulting in the accumulation of α-neurite nuclein (Luo et al., 2019). RGS6 has an important effect on the differentiation of microtubules and neurons. RGS6 induces neuron differentiation through a new mechanism involving the interaction of SCG10 with its GGL domain (Liu et al., 2002). Existing studies are consistent with our analysis results. RGS6 is expressed in humans and mice and is regarded as an important characteristic gene in cerebellar cells. Compared with the existing research, our analysis locates the specific cell type of the RGS6 expression site from the single-cell level, laying a foundation for in-depth mechanism research.

### Analysis of Combined-Gene Rules

We built total 23 combined-gene rules through the DT method. A total of 8, 2, 2 and 11 decision rules are observed for distinguishing human from mouse cerebellum in Golgi cell, GC, interneuron cell, and UBC, respectively. Given the biological significance of these traits, how do we determine the stage of evolution and development on the basis of the expression of these traits? Here are some studies to introduce their experimental evidence.

### Golgi Cell Rules

The protein coding gene *LINGO2* is one of the four important families related to the nervous system (LINGO1–4) (Llorens et al., 2008; Homma et al., 2009). This gene is enriched in early spermatids, late spermatids, and bipolar cells. The *LINGO2* expression is detected in the neuronal tissues of the brains of adult mice (Vilarino-Guell et al., 2010). The *LINGO2* variant detected in the Chinese population may increase the risk of gestational diabetes. The results of a large number of Asian population studies showed that *LINGO2* may be a susceptibility gene for ET and Parkinson’s syndrome and that the increased expression of LINGO is a characteristic pathological response of neurodegenerative diseases (Delay et al., 2014). When the expression of LINGO2 gene is high, a low degree of nervous system evolution is observed. By contrast, the expression of the LINGO gene is low when the species evolves at a high level especially when the nervous system is developed. On the basis of existing research and our analysis results, we speculate that LINGO2 may show specific expression patterns at different stages of neurodevelopment and species evolution, which may provide references for interpreting neurological disorders.

Thsd7b is another important gene involved in the decision rules by our analysis. The thrombospondin type 1 domain containing 7B (Thsd7b) is a protein-coding gene. Thsd7b is enriched in human brain regions especially the pons and medulla. Thsd7b is closely related to O-linked glycosylation, glycosylation diseases, and Ehlers–Danlos syndrome. Researchers found a correlation between Thsd7b and the formation of cisplatin resistance. A large number of studies on pancreatic cancer in Japan showed that the Thsd7b gene is significantly associated with the risk of pancreatic cancer. In addition, this gene is related to the prognosis of non-small cell lung cancer with chemotherapy intervention (Lee et al., 2013). At present, no study is available on the mechanism of Thsd7b related to the cerebellum. Our analysis results fill in the gaps in the development of the cerebellum especially the evolution of the Golgi cell.

#### Granule Cell and Interneuron Cell Rule

The RNA coding gene Malat1 belongs to the long noncoding RNA (lncRNA) category. lncRNA is closely related to diseases, such as stroke (Qureshi and Mehler, 2012) and ischemic stroke (Zhang et al., 2017). Malat1 is highly conserved, and previous studies found that Malat1 is closely related to diseases, such as hyperglycemia, leukemia, and acute mononucleosis. Recent research results revealed that Malat1 is related to the metastasis of lung cancer cells (Ji et al., 2003). In addition, Malat1 promotes the development of renal carcinoma by interacting with Ezh2 (Hirata et al., 2015). Tumor cell proliferation in esophageal cancer is suppressed by Malat1 silencing (Wang et al., 2015). Studies reported that Malat1 can be involved in regulating the function of endothelial cells and the growth of blood vessels (Michalik et al., 2014). The downregulation of Malat1 expression promotes the macrophage polarization to the M1 phenotype. Our analysis results showed that when Malat1 is expressed at high levels in GCs and interneuron cells, the nervous system becomes mature. Interestingly, the siRNA-mediated downregulation of Malat1 promotes T cell proliferation and accelerates the transformation of T cells into the Th1/Th17 cell spectrum. The contribution of Malat1 lncRNA to autoimmune neuroinflammation has been observed in patients with multiple sclerosis and mice with encephalomyelitis (Masoumi et al., 2019). These data indicate that Malat1 has a potential anti-inflammatory effect in the context of autoimmune neuroinflammation. The regulatory mechanism of Malat1 helps to ascertain the therapeutic targets of central nervous system diseases and help to establish a complete treatment strategy.

### Unipolar Brush Cell Rule

A relatively higher expression of Ccdc85a in UBCs was required to indicate human cerebellum in decision rules. The protein-coding gene coiled-coil domain containing 85A (Ccdc85a) is expressed in various brain regions especially in the cerebral cortex. Diseases associated with Ccdc85a include hydrocephalus. Studies confirmed that the Ccdc85a protein is required in the AppNL-F interaction group. The results of colocalization analysis indicate that the Ccdc85a protein may endogenously regulate the function of the amyloid β-protein. In the early stage of Alzheimer’s disease, the upregulation of Ccdc85a expression may be a compensation for the increase in amyloid β-protein and the elimination of amyloid β-protein metabolism. These findings suggest that Ccdc85a may play an important role in nervous system development and evolution, and become a new target and biomarker for clinical intervention in neurological dysfunction diseases (Aladeokin et al., 2019).

We use the mRMR feature screening method to conduct an in-depth analysis of the existing single-cell transcriptome data set and select the key and characteristically expressed genes. Subsequently, we use DT and SMOTE tools to determine the expression rules of characteristic genes. In the end, we obtain key genes that may be related to evolution and neurodevelopment and confirm the decision-making rules which reflect the heterogeneity between species in different cerebellar cell types. Our analysis results are consistent with many existing research conclusions, but the specific pathogenic molecular mechanism of each characteristic gene needs further verification. Overall, this research has obtained representative species evolution genes and their expression differences in various cerebellar cells. The remarkable potential of these features and rules in studying species evolution and are highlighted and provide insights into new key genes. The excellent performance of our classifiers can be attributed to the strong specificity of gene expression at species and tissue levels, which can significantly distinguish human and mouse. In addition, our research methods and strategies have a good guiding role in exploring genetic evolution. Therefore, the characteristic genes we have identified can be used to identify specific cell groups and the evolutionary level of species and can be regarded as biological indicators to provide research directions for disease-related molecular mechanisms.

## Data Availability

Publicly available datasets were analyzed in this study. This data can be found here: https://www.ncbi.nlm.nih.gov/geo/query/acc.cgi?acc=GSE165371.

## References

[B112] Ahlers K. E. Chakravarti B. Fisher R. a.-O. (2016). RGS6 as a Novel Therapeutic Target in CNS Diseases and Cancer. AAPS J. 18, 560–572. 27002730 10.1208/s12248-016-9899-9 PMC5256616

[B1] Aladeokin A. C. Akiyama T. Kimura A. Kimura Y. Takahashi-Jitsuki A. Nakamura H. (2019). Network-guided Analysis of Hippocampal Proteome Identifies Novel Proteins that Colocalize with Aβ in a Mice Model of Early-Stage Alzheimer's Disease. Neurobiol. Dis. 132, 104603. 10.1016/j.nbd.2019.104603 31494281

[B2] Banerjee S. Neveu P. Kosik K. S. (2009). A Coordinated Local Translational Control point at the Synapse Involving Relief from Silencing and MOV10 Degradation. Neuron 64, 871–884. 10.1016/j.neuron.2009.11.023 20064393

[B3] Bifsha P. Yang J. Fisher R. A. Drouin J. (2014). Rgs6 Is Required for Adult Maintenance of Dopaminergic Neurons in the Ventral Substantia Nigra. Plos Genet. 10, e1004863. 10.1371/journal.pgen.1004863 25501001 PMC4263397

[B4] Bird T. D. Adam M. P. Ardinger H. H. Pagon R. A. Wallace S. E. Bean L. J. H. (1993). “GDAP1-Related Hereditary Motor and Sensory Neuropathy,” in GeneReviews((R)) (Bethesda, MD, United States: Seattle). 20301711

[B5] Breiman L. (2001). Random Forests. Machine Learn. 45, 5–32. 10.1023/a:1010933404324

[B95] Buiting K. Williams C. Horsthemke B. (2016). Angelman Syndrome-Insights into a Rare Neurogenetic Disorder. Nat. Rev. Neurol. 12, 584–593. 27615419 10.1038/nrneurol.2016.133

[B6] Chawla N. V. Bowyer K. W. Hall L. O. Kegelmeyer W. P. (2002). SMOTE: Synthetic Minority Over-sampling Technique. jair 16, 321–357. 10.1613/jair.953

[B7] Chen L. Li Z. Zhang S. Zhang Y.-H. Huang T. Cai Y.-D. (2022). Predicting RNA 5-methylcytosine Sites by Using Essential Sequence Features and Distributions. Biomed. Res. Int. 10.1155/2022/4035462 PMC8776474 35071593

[B8] Chen L. Wang S. Zhang Y.-H. Li J. Xing Z.-H. Yang J. (2017). Identify Key Sequence Features to Improve CRISPR sgRNA Efficacy. IEEE Access 5, 26582–26590. 10.1109/access.2017.2775703

[B9] Chen S. Krinsky B. H. Long M. (2013). New Genes as Drivers of Phenotypic Evolution. Nat. Rev. Genet. 14 (9), 645–660. 10.1038/nrg3521 23949544 PMC4236023

[B115] Chen Y. Cao B. Yang J. Wei Q. Ou R. W. Zhao B. Song W. (2015). Analysis and Meta-Analysis of Five Polymorphisms of the LINGO1 and LINGO2 Genes in Parkinson's Disease and Multiple System Atrophy in a Chinese Population. J. Neurol. 262, 2478–2483. 26254004 10.1007/s00415-015-7870-9

[B10] Chen W. Chen L. Dai Q. (2021). iMPT-FDNPL: Identification of Membrane Protein Types with Functional Domains and a Natural Language Processing Approach. Comput. Math. Methods Med. 2021, 7681497. 10.1155/2021/7681497 34671418 PMC8523280

[B11] Clapéron A. Hattab C. Armand V. Trottier S. Bertrand O. Ouimet T. (2007). The Kell and XK Proteins of the Kell Blood Group Are Not Co-expressed in the central Nervous System. Brain Res. 1147, 12–24. 10.1016/j.brainres.2007.01.106 17379193

[B12] Cooper D. N. Kehrer-Sawatzki H. (2011). Exploring the Potential Relevance of Human-specific Genes to Complex Disease. Hum. Genomics 5 (2), 99–107. 10.1186/1479-7364-5-2-99 21296743 PMC3525227

[B13] D'angelo E. Casali S. (2012). Seeking a Unified Framework for Cerebellar Function and Dysfunction: from Circuit Operations to Cognition. Front. Neural Circuits 6, 116. 10.3389/fncir.2012.00116 23335884 PMC3541516

[B14] D'angelo E. De Zeeuw C. I. (2009). Timing and Plasticity in the Cerebellum: Focus on the Granular Layer. Trends Neurosci. 32, 30–40. 10.1016/j.tins.2008.09.007 18977038

[B15] D'angelo E. (2010). Neuronal Circuit Function and Dysfunction in the Cerebellum: from Neurons to Integrated Control. Funct. Neurol. 25, 125–127. 21232207

[B16] D'angelo E. (2011). Neural Circuits of the Cerebellum: Hypothesis for Function. J. Integr. Neurosci. 10, 317–352. 10.1142/s0219635211002762 21960306

[B17] D'angelo E. (2018). Physiology of the Cerebellum. Handb Clin. Neurol. 154, 85–108. 10.1016/b978-0-444-63956-1.00006-0 29903454

[B18] Danek A. Rubio J. P. Rampoldi L. Ho M. Dobson-Stone C. Tison F. o. (2001). McLeod Neuroacanthocytosis: Genotype and Phenotype. Ann. Neurol. 50, 755–764. 10.1002/ana.10035 11761473

[B19] Danek A. Walker R. H. (2005). Neuroacanthocytosis. Curr. Opin. Neurol. 18, 386–392. 10.1097/01.wco.0000173464.01888.e9 16003113

[B88] Delay C. Tremblay C. Brochu E. Paris-Robidas S. Emond V. Rajput A. H. (2014). Increased LINGO1 in the Cerebellum of Essential Tremor Patients. Mov. Disord. 29, 1637–1647. 24531928 10.1002/mds.25819

[B20] De Zeeuw P. Van Belle J. Van Dijk S. Weusten J. Koeleman B. Janson E. (2012). Imaging Gene and Environmental Effects on Cerebellum in Attention-Deficit/Hyperactivity Disorder and Typical Development. Neuroimage Clin. 2, 103–110. 10.1016/j.nicl.2012.11.010 24179763 PMC3777835

[B21] Delay C. Tremblay C. Brochu E. Paris-Robidas S. Emond V. Rajput A. H. (2014). Increased LINGO1 in the Cerebellum of Essential Tremor Patients. Mov Disord. 29, 1637–1647. 10.1002/mds.25819 24531928

[B90] Dhananjayan S. C. Ramamoorthy S. Khan O. Y. Ismail A. Sun J. Slingerland J. (2006). WW Domain Binding Protein-2, An E6-Associated Protein Interacting Protein, Acts As A Coactivator of Estrogen and Progesterone Receptors. Mol. Endocrinol. 20, 2343–2354. 16772533 10.1210/me.2005-0533

[B22] Ding S. Wang D. Zhou X. Chen L. Feng K. Xu X. (2022). Predicting Heart Cell Types by Using Transcriptome Profiles and a Machine Learning Method. Life 12, 228. 10.3390/life12020228 35207515 PMC8877019

[B23] Diño M. R. Mugnaini E. (2000). Postsynaptic Actin Filaments at the Giant Mossy Fiber-Unipolar brush Cell Synapse. Synapse 38, 499–510. 10.1002/1098-2396(20001215)38:4<499::AID-SYN16>3.0.CO;2-H 11044898

[B24] Diño M. R. Perachio A. A. Mugnaini E. (2001). Cerebellar Unipolar brush Cells Are Targets of Primary Vestibular Afferents: an Experimental Study in the Gerbil. Exp. Brain Res. 140, 162–170. 10.1007/s002210100790 11521148

[B25] Eccles J. C. Llinás R. Sasaki K. (1966). The Mossy Fibre-Granule Cell Relay of the Cerebellum and its Inhibitory Control by Golgi Cells. Exp. Brain Res. 1 (1), 82–101. 10.1007/BF00235211 5910945

[B26] Engel T. Kannenberg F. Fobker M. Nofer J.-R. Bode G. Lueken A. (2007). Expression of ATP Binding Cassette-Transporter ABCG1 Prevents Cell Death by Transporting Cytotoxic 7β-Hydroxycholesterol. FEBS Lett. 581, 1673–1680. 10.1016/j.febslet.2007.03.038 17408620

[B27] Fijal B. A. Stauffer V. L. Kinon B. J. Conley R. R. Hoffmann V. P. Witte M. M. (2012). Analysis of Gene Variants Previously Associated with Iloperidone Response in Patients with Schizophrenia Who Are Treated with Risperidone. J. Clin. Psychiatry 73, 367–371. 10.4088/jcp.10m06507 21813073

[B28] Fukushima K. Pollock D. D. (2020). Amalgamated Cross-Species Transcriptomes Reveal Organ-specific Propensity in Gene Expression Evolution. Nat. Commun. 11, 4459. 10.1038/s41467-020-18090-8 32900997 PMC7479108

[B93] Gossan N. C. Zhang F. Guo B. Q. Jin D. Yoshitane H. Yao A. Y. (2014). The E3 Ubiquitin Ligase UBE3A is an Integral Component of the Molecular Circadian Clock through Regulating the BMAL1 Transcription Factor. Nucl. Acids Res. 42, 5765–5775. 24728990 10.1093/nar/gku225 PMC4027211

[B96] Greer P. L. Hanayama R. Bloodgood B. L. Mardinly A. R. Lipton D. M. Flavell S. W. (2010). The Angelman Syndrome Protein Ube3A Regulates Synapse Development by Ubiquitinating Arc. Cell 140, 704–716. 20211139 10.1016/j.cell.2010.01.026 PMC2843143

[B110] Gessner G. Runge S. Koenen M. Heinemann S. H. Koenen M. Haas J. (2019). ANK2 Functionally Interacts with KCNH2 Aggravating Long QT Syndrome in a Double Mutation Carrier. Biochem. Biophys. Res. Commun. 512, 845–851. 30929919 10.1016/j.bbrc.2019.03.162

[B29] Hanchuan Peng H. Fuhui Long F. Ding C. (2005). Feature Selection Based on Mutual Information Criteria of max-dependency, max-relevance, and Min-Redundancy. IEEE Trans. Pattern Anal. Machine Intell. 27, 1226–1238. 10.1109/tpami.2005.159 16119262

[B30] Hansel C. Linden D. J. D'angelo E. (2001). Beyond Parallel Fiber LTD: the Diversity of Synaptic and Non-synaptic Plasticity in the Cerebellum. Nat. Neurosci. 4, 467–475. 10.1038/87419 11319554

[B31] Hirata H. Hinoda Y. Shahryari V. Deng G. Nakajima K. Tabatabai Z. L. (2015). Long Noncoding RNA MALAT1 Promotes Aggressive Renal Cell Carcinoma through Ezh2 and Interacts with miR-205. Cancer Res. 75, 1322–1331. 10.1158/0008-5472.can-14-2931 25600645 PMC5884967

[B32] Homma S. Shimada T. Hikake T. Yaginuma H. (2009). Expression Pattern of LRR and Ig Domain-Containing Protein (LRRIG Protein) in the Early Mouse Embryo. Gene Expr. Patterns 9, 1–26. 10.1016/j.gep.2008.09.004 18848646

[B102] Hoxha E. Marcinno A. Montarolo F. Masante L. Balbo I. Ravera F. (2019). Emerging Roles of Fgf14 in Behavioral Control. Behav. Brain Res. 356, 257–265. 30189289 10.1016/j.bbr.2018.08.034 PMC10082543

[B33] Huang Y. Liao X. Luo J. Liu H. Zhong S. Chen J. (2020). Expression of Circular RNAs in the Vascular Dementia Rats. Neurosci. Lett. 735, 135087. 10.1016/j.neulet.2020.135087 32534097

[B34] Ichikawa M. Aiba T. Ohno S. Shigemizu D. Ozawa J. Sonoda K. (2016). Phenotypic Variability of ANK2 Mutations in Patients with Inherited Primary Arrhythmia Syndromes. Circ. J. 80, 2435–2442. 10.1253/circj.cj-16-0486 27784853

[B35] Jaarsma D. Diño M. R. Cozzari C. Mugnaini E. (1996). Cerebellar Choline Acetyltransferase Positive Mossy Fibres and Their Granule and Unipolar brush Cell Targets: A Model for central Cholinergic Nicotinic Neurotransmission. J. Neurocytol 25, 829–842. 10.1007/bf02284845 9023728

[B87] Jasinska-Myga B. Wider C. (2012). Genetics of Essential Tremor. Parkinsonism Relat. Disord. 18, S138–S139. 22166413 10.1016/S1353-8020(11)70043-8

[B97] Ji C. N. Chen J. Z. Xie Y. Wang S. Qian J. Zhao E. (2003a). A Novel cDNA Encodes a Putative hRALY-Like Protein, hRALYL. Mol. Biol. Rep. 30, 61–67. 12688537 10.1023/a:1022295311177

[B36] Ji P. Diederichs S. Wang W. Böing S. Metzger R. Schneider P. M. (2003). MALAT-1, a Novel Noncoding RNA, and Thymosin β4 Predict Metastasis and Survival in Early-Stage Non-small Cell Lung Cancer. Oncogene 22, 8031–8041. 10.1038/sj.onc.1206928 12970751

[B37] Jia Y. Zhao R. Chen L. (2020). Similarity-Based Machine Learning Model for Predicting the Metabolic Pathways of Compounds. IEEE Access 8, 130687–130696. 10.1109/access.2020.3009439

[B38] Jung H. H. Hergersberg M. Kneifel S. Alkadhi H. Schiess R. Weigell-Weber M. (2001). McLeod Syndrome: a Novel Mutation, Predominant Psychiatric Manifestations, and Distinct Striatal Imaging Findings. Ann. Neurol. 49, 384–392. 10.1002/ana.76 11261514

[B39] Kaessmann H. (2010). Origins, Evolution, and Phenotypic Impact of New Genes. Genome Res. 20, 1313–1326. 10.1101/gr.101386.109 20651121 PMC2945180

[B40] Kamat S. S. Camara K. Parsons W. H. Chen D.-H. Dix M. M. Bird T. D. (2015). Immunomodulatory Lysophosphatidylserines Are Regulated by ABHD16A and ABHD12 Interplay. Nat. Chem. Biol. 11, 164–171. 10.1038/nchembio.1721 25580854 PMC4301979

[B41] Karten B. Campenot R. B. Vance D. E. Vance J. E. (2006). Expression of ABCG1, but Not ABCA1, Correlates with Cholesterol Release by Cerebellar Astroglia. J. Biol. Chem. 281, 4049–4057. 10.1074/jbc.m508915200 16352604

[B111] Kelly M. P. Adamowicz W. Bove S. Hartman A. J. Mariga A. Pathak G. (2014). Select 3',5'-Cyclic Nucleotide Phosphodiesterases Exhibit Altered Expression in the Aged Rodent Brain. Cell Signal 26, 383–397. 24184653 10.1016/j.cellsig.2013.10.007

[B42] Kepecs A. Fishell G. (2014). Interneuron Cell Types Are Fit to Function. Nature 505, 318–326. 10.1038/nature12983 24429630 PMC4349583

[B104] Keum S. Kim A. Shin J. J. Kim J. H. Park J. Shin H. S. (2018). A Missense Variant at the Nrxn3 Locus Enhances Empathy Fear in the Mouse. Neuron 98, 588–601. 29681532 10.1016/j.neuron.2018.03.041

[B43] Kobayashi A. Takanezawa Y. Hirata T. Shimizu Y. Misasa K. Kioka N. (2006). Efflux of Sphingomyelin, Cholesterol, and Phosphatidylcholine by ABCG1. J. Lipid Res. 47, 1791–1802. 10.1194/jlr.m500546-jlr200 16702602

[B44] Kohavi R. (1995). “A Study of Cross-Validation and Bootstrap for Accuracy Estimation and Model Selection,” in International Joint Conference on Artificial Intelligence (Lawrence Erlbaum Associates), 1137–1145.

[B89] Kumar S. Talis A. L. Howley P. M. (1999). Identification of HHR23A as a Substrate for E6-Associated Protein-Mediated Ubiquitination. J. Biol. Chem. 274, 18785–18792. 10373495 10.1074/jbc.274.26.18785

[B45] Kursa M. B. Rudnicki W. R. (2010). Feature Selection with the Boruta Package. J. Stat. Softw. 36, 1–13. 10.18637/jss.v036.i11

[B46] Lantieri F. Glessner J. T. Hakonarson H. Elia J. Devoto M. (2010). Analysis of GWAS Top Hits in ADHD Suggests Association to Two Polymorphisms Located in Genes Expressed in the Cerebellum. Am. J. Med. Genet. B Neuropsychiatr. Genet. 153B, 1127–1133. 10.1002/ajmg.b.31110 20607790

[B47] Lavedan C. Licamele L. Volpi S. Hamilton J. Heaton C. Mack K. (2009). Association of the NPAS3 Gene and Five Other Loci with Response to the Antipsychotic Iloperidone Identified in a Whole Genome Association Study. Mol. Psychiatry 14, 804–819. 10.1038/mp.2008.56 18521090

[B48] Lee S. Sha Q. Wu X. Calenda G. Peng J. (2007). Expression Profiles of Mouse Kell, XK, and XPLAC mRNA. J. Histochem. Cytochem. 55, 365–374. 10.1369/jhc.6a7126.2006 17189525

[B49] Lee Y. Yoon K.-A. Joo J. Lee D. Bae K. Han J.-Y. (2013). Prognostic Implications of Genetic Variants in Advanced Non-small Cell Lung Cancer: a Genome-wide Association Study. Carcinogenesis 34, 307–313. 10.1093/carcin/bgs356 23144319

[B50] Li X. Lu L. Chen L. (2022). Identification of Protein Functions in Mouse with a Label Space Partition Method. Math. biosciences Eng. 10.3934/mbe.2022176 35341276

[B114] Liu Z. Chatterjee T. K. Fisher R. A. (2002). RGS6 Interacts with SCG10 and Promotes Neuronal Differentiation. Role of the G Gamma Subunit-Like (GGL) Domain of RGS6. J. Biol. Chem. 277, 37832–37839. 12140291 10.1074/jbc.M205908200

[B51] Liang H. Chen L. Zhao X. Zhang X. (2020). Prediction of Drug Side Effects with a Refined Negative Sample Selection Strategy. Comput. Math. Methods Med. 2020, 1573543. 10.1155/2020/1573543 32454877 PMC7232712

[B52] Liu H. Hu B. Chen L. Lu L. (2021). Identifying Protein Subcellular Location with Embedding Features Learned from Networks. Cp 18, 646–660. 10.2174/1570164617999201124142950

[B53] Liu H. Setiono R. (1998). Incremental Feature Selection. Appl. Intelligence 9, 217–230. 10.1023/a:1008363719778

[B113] Luo Z. Ahlers-Dannen K. E. Spicer M. M. Yang J. Alberico S. Stevens H. E. (2019). Age-Dependent Nigral Dopaminergic Neurodegeneration and ℵ-Synuclein Accumulation in RGS6-Deficient Mice. JCI Insight 5, e126769. 10.1172/jci.insight.126769 PMC6629243 31120439

[B54] Llorens F. Gil V. Iraola S. Carim-Todd L. Martí E. Estivill X. (2008). Developmental Analysis of Lingo-1/Lern1 Protein Expression in the Mouse Brain: Interaction of its Intracellular Domain with Myt1l. Devel Neurobio 68, 521–541. 10.1002/dneu.20607 18186492

[B55] Mahley R. W. (2016). Central Nervous System Lipoproteins. Atvb 36, 1305–1315. 10.1161/atvbaha.116.307023 PMC4942259 27174096

[B56] Makide K. Uwamizu A. Shinjo Y. Ishiguro J. Okutani M. Inoue A. (2014). Novel Lysophosphoplipid Receptors: Their Structure and Function. J. Lipid Res. 55, 1986–1995. 10.1194/jlr.r046920 24891334 PMC4173991

[B57] Masoumi F. Ghorbani S. Talebi F. Branton W. G. Rajaei S. Power C. (2019). Malat1 Long Noncoding RNA Regulates Inflammation and Leukocyte Differentiation in Experimental Autoimmune Encephalomyelitis. J. Neuroimmunology 328, 50–59. 10.1016/j.jneuroim.2018.11.013 30583215

[B58] Matthews B. W. (1975). Comparison of the Predicted and Observed Secondary Structure of T4 Phage Lysozyme. Biochim. Biophys. Acta (Bba) - Protein Struct. 405, 442–451. 10.1016/0005-2795(75)90109-9 1180967

[B59] Michalik K. M. You X. Manavski Y. Doddaballapur A. Zörnig M. Braun T. (2014). Long Noncoding RNA MALAT1 Regulates Endothelial Cell Function and Vessel Growth. Circ. Res. 114, 1389–1397. 10.1161/circresaha.114.303265 24602777

[B91] Mishra A. Godavarthi S. K. Maheshwari M. Goswami A. Jana N. R. (2009). The Ubiquitin Ligase E6-AP Is Induced and Recruited to Aggresomes in Response to Proteasome Inhibition and May Be Involved in the Ubiquitination of Hsp70-Bound Misfolded Proteins. J. Biol. Chem. 129, 611–615. 10.1074/jbc.M806804200 PMC2667740 19233847

[B60] Neale B. M. Lasky-Su J. Anney R. Franke B. Zhou K. Maller J. B. (2008). Genome-wide Association Scan of Attention Deficit Hyperactivity Disorder. Am. J. Med. Genet. 147B, 1337–1344. 10.1002/ajmg.b.30866 18980221 PMC2831205

[B108] Novak G. Boukhadra J. Shaikh S. A. Kennedy J. L. Le Foll B. (2009). Association of a Polymorphism in the NRXN3 Gene with the Degree of Smoking in Schizophrenia: A Preliminary Study. World J. Biol. Psychiatry 10, 929–935. 19658047 10.1080/15622970903079499

[B61] Oertel D. Young E. D. (2004). What's a Cerebellar Circuit Doing in the Auditory System? Trends Neurosciences 27, 104–110. 10.1016/j.tins.2003.12.001 15102490

[B62] Pan X. Li H. Zeng T. Li Z. Chen L. Huang T. (2021). Identification of Protein Subcellular Localization with Network and Functional Embeddings. Front. Genet. 11, 626500. 10.3389/fgene.2020.626500 33584818 PMC7873866

[B103] Pablo J. L. Pitt G. S. (2017). FGF14 is a Regulator of KCNQ2/3 Channels. Proc. Natl. Acad. Sci. U S A 114, 154–159. 27994149 10.1073/pnas.1610158114 PMC5224356

[B63] Qureshi I. A. Mehler M. F. (2012). Emerging Roles of Non-coding RNAs in Brain Evolution, Development, Plasticity and Disease. Nat. Rev. Neurosci. 13, 528–541. 10.1038/nrn3234 22814587 PMC3478095

[B64] Raudenská M. Bittnerová A. Novotný T. Floriánová A. Chroust K. Gaillyová R. (2008). Mutation Analysis of Candidate Genes SCN1B, KCND3 and ANK2 in Patients with Clinical Diagnosis of Long QT Syndrome. Physiol. Res. 57, 857–862. 10.33549/physiolres.931184 18052691

[B94] Ronchi V. P. Klein J. M. Edwards D. J. Haas A. L. (2014). The Active Form of E6-Associated Protein (E6AP)/UBE3A Ubiquitin Ligase Is an Oligomer. J. Biol. Chem. 289, 1033–1048. 24273172 10.1074/jbc.M113.517805 PMC3887172

[B65] Safavian S. R. Landgrebe D. (1991). A Survey of Decision Tree Classifier Methodology. IEEE Trans. Syst. Man. Cybern. 21, 660–674. 10.1109/21.97458

[B66] Sekerková G. Ilijic E. Mugnaini E. Baker J. F. (2005). Otolith Organ or Semicircular Canal Stimulation Induces C-Fos Expression in Unipolar brush Cells and Granule Cells of Cat and Squirrel Monkey. Exp. Brain Res. 164, 286–300. 10.1007/s00221-005-2252-7 15940501

[B67] Shah M. M. Mistry M. Marsh S. J. Brown D. A. Delmas P. (2002). Molecular Correlates of the M‐current in Cultured Rat Hippocampal Neurons. J. Physiol. 544, 29–37. 10.1113/jphysiol.2002.028571 12356878 PMC2290582

[B68] Shook D. Brouwer R. De Zeeuw P. Oranje B. Durston S. (2017). XKR4 Gene Effects on Cerebellar Development Are Not Specific to ADHD. Front. Cel. Neurosci. 11, 396. 10.3389/fncel.2017.00396 PMC5732973 29311829

[B92] Shimoji T. Murakami K Fau-Sugiyama Y. Sugiyama Y Fau-Matsuda M. Y. Matsuda M Fau-Inubushi S. Inubushi S Fau-Nasu J. Nasu J Fau-Shirakura M. (2009). Identification of Annexin A1 as a Novel Substrate for E6AP-Mediated Ubiquitylation. J Cell Biochem 106, 1123–1135. 19204938 10.1002/jcb.22096

[B69] Strick P. L. (1985). The Cerebellum: The Cerebellum and Neural Control . Masao Ito. Raven, New York, 1984. Xviii, 580 pp., Illus. $75. Science 229, 547. 10.1126/science.229.4713.547 17732433

[B106] Südhof T. C. (2008). Neuroligins and Neurexins Link Synaptic Function to Cognitive Disease. Nature 455, 903–911. 18923512 10.1038/nature07456 PMC2673233

[B70] Tachikawa M. Toki H. Watanabe M. Tomi M. Hosoya K.-i. Terasaki T. (2018). Gene Expression of A6-like Subgroup of ATP-Binding Cassette Transporters in Mouse Brain Parenchyma and Microvessels. Anat. Sci. Int. 93, 456–463. 10.1007/s12565-018-0435-0 29520568

[B71] Tang S. Chen L. (2022). iATC-NFMLP: Identifying Classes of Anatomical Therapeutic Chemicals Based on Drug Networks, Fingerprints and Multilayer Perceptron. Curr. Bioinformatics.

[B72] Uhl G. R. Drgon T. Johnson C. Fatusin O. O. Liu Q.-R. Contoreggi C. (2008). "Higher Order" Addiction Molecular Genetics: Convergent Data from Genome-wide Association in Humans and Mice. Biochem. Pharmacol. 75, 98–111. 10.1016/j.bcp.2007.06.042 17764662 PMC3282179

[B73] Vilariño-Güell C. Wider C. Ross O. A. Jasinska-Myga B. Kachergus J. Cobb S. A. (2010). LINGO1 and LINGO2 Variants Are Associated with Essential Tremor and Parkinson Disease. Neurogenetics 11, 401–408. 10.1007/s10048-010-0241-x 20369371 PMC3930084

[B74] Wang F. Gu H.-m. Zhang D.-w. (2014). Caveolin-1 and ATP Binding Cassette Transporter A1 and G1-Mediated Cholesterol Efflux. Chddt 14, 142–148. 10.2174/1871529x14666140505122802 24801727

[B109] Watanabe H. Minamino T. (2016). Rare Variants in ANK2 Associated With Various Inherited Arrhythmia Syndromes. Circ. J. 80, 2423–2424. 27818464 10.1253/circj.CJ-16-1085

[B75] Wang X. Li M. Wang Z. Han S. Tang X. Ge Y. (2015). Silencing of Long Noncoding RNA MALAT1 by miR-101 and miR-217 Inhibits Proliferation, Migration, and Invasion of Esophageal Squamous Cell Carcinoma Cells. J. Biol. Chem. 290, 3925–3935. 10.1074/jbc.m114.596866 25538231 PMC4326802

[B86] Wu Y. W. Prakash K. M. Rong T. Y. Li H. H. Xiao Q. Tan L. C. (2011). Lingo2 Variants Associated with Essential Tremor and Parkinson's Disease. Hum. Genet. 129, 611–615. 21287203 10.1007/s00439-011-0955-3

[B99] Wang X. Wang J. Tsui Y. M. Shi C. Wang Y. Zhang X. (2021). RALYL Increases Hepatocellular Carcinoma Stemness by Sustaining the mRNA Stability of TGF-β2. Nat. Commun. 12, 1518. 33750796 10.1038/s41467-021-21828-7 PMC7943813

[B100] Xia Y. Ye S. Yang Y. Liu Y. Tong G. (2021). Over-Expression of RALYL Suppresses the Progression of Ovarian Clear Cell Carcinoma through Inhibiting MAPK and CDH1 Signaling Pathways. Int. J. Med. Sci. 18, 785–791. 33437214 10.7150/ijms.51488 PMC7797558

[B76] Yang Y. Chen L. (2022). Identification of Drug-Disease Associations by Using Multiple Drug and Disease Networks. Cbio 17, 48–59. 10.2174/1574893616666210825115406

[B101] Miura S. Kosaka K. Fujioka R. Uchiyama Y. Shimojo T. Morikawa T. (2019). Spinocerebellar ataxia 27 with a Novel Nonsense Variant (Lys177X) in FGF14. Eur. J. Med. Genet. 62, 172–176. 30017992 10.1016/j.ejmg.2018.07.005

[B107] Yuan H. Wang Q. Liu Y. Yang W. He Y. Gusella J. F. (2018). A Rare Exonic NRXN3 Deletion Segregating with Neurodevelopmental and Neuropsychiatric Conditions in a Three-Generation Chinese Family. Am. J. Med. Genet. B Neuropsychiatr. Genet. 177, 589–595. 30076746 10.1002/ajmg.b.32673 PMC6445570

[B77] Zhang X. Tang X. Liu K. Hamblin M. H. Yin K.-J. (2017). Long Noncoding RNA Malat1 Regulates Cerebrovascular Pathologies in Ischemic Stroke. J. Neurosci. 37, 1797–1806. 10.1523/jneurosci.3389-16.2017 28093478 PMC5320610

[B98] Zhang Y. Wang J. Liu X. Liu H. (2020). Exploring the Role of RALYL in Alzheimer's Disease Reserve by Network-Based Approaches. Alzheimers Res. Ther. 12, 165. 33298176 10.1186/s13195-020-00733-z PMC7724892

[B78] Zhang Y. Zhu H. Zhao L. Zhou X. Huang P. (2008). Generation of Mouse UBE2W Antibody and Analysis of UBE2W Expression in Mouse Tissues. Sheng Wu Gong Cheng Xue Bao 24, 547–552. 18616160

[B79] Zhang Y.-H. Li H. Zeng T. Chen L. Li Z. Huang T. (2021a). Identifying Transcriptomic Signatures and Rules for SARS-CoV-2 Infection. Front. Cel Dev. Biol. 8, 627302. 10.3389/fcell.2020.627302 PMC7829664 33505977

[B80] Zhang Y.-H. Zeng T. Chen L. Huang T. Cai Y.-D. (2021b). Determining Protein-Protein Functional Associations by Functional Rules Based on Gene Ontology and KEGG Pathway. Biochim. Biophys. Acta (Bba) - Proteins Proteomics 1869, 140621. 10.1016/j.bbapap.2021.140621 33561576

[B81] Zhang Y. E. Landback P. Vibranovski M. D. Long M. (2011). Accelerated Recruitment of New Brain Development Genes into the Human Genome. Plos Biol. 9 (10), e1001179. 10.1371/journal.pbio.1001179 22028629 PMC3196496

[B82] Zhang Y. E. Long M. (2014). New Genes Contribute to Genetic and Phenotypic Novelties in Human Evolution. Curr. Opin. Genet. Dev. 29, 90–96. 10.1016/j.gde.2014.08.013 25218862 PMC4631527

[B83] Zhao B. Cui Y. Fan X. Qi P. Liu C. Zhou X. (2019). Anti-obesity Effects of Spirulina Platensis Protein Hydrolysate by Modulating Brain-Liver axis in High-Fat Diet Fed Mice. PLoS One 14, e0218543. 10.1371/journal.pone.0218543 31220177 PMC6586325

[B84] Zhao X. Chen L. Lu J. (2018). A Similarity-Based Method for Prediction of Drug Side Effects with Heterogeneous Information. Math. Biosciences 306, 136–144. 10.1016/j.mbs.2018.09.010 30296417

[B105] Zheng J. J. Li Wx Fau-Liu J.-Q. Liu Jq Fau-Guo Y.-C. Guo Yc Fau-Wang Q. Wang Q Fau-Li G.-H. Li Gh Fau-Dai S.-X. (2018). Low Expression of Aging-Related NRXN3 is Associated with Alzheimer Disease: A Systematic Review and Meta-Analysis. Medicine (Baltimore) 97. 10.1097/MD.0000000000011343 PMC6076205 29995770

[B85] Zili Luo K. E. A. Yang J. Chakravarti B. Stevens H. E. Nandakumar S. N. Fisher R. a. (2018). Regulator of G-Protein Signaling 6 (RGS6) Expression in Human Substantia Nigra Pars Compacta (SNc) and Loss in Parkinson's Disease (PD). FASEB J. 31, 659623–659659.

